# Safety and Immunogenicity of rSh28GST Antigen in Humans: Phase 1 Randomized Clinical Study of a Vaccine Candidate against Urinary Schistosomiasis

**DOI:** 10.1371/journal.pntd.0001704

**Published:** 2012-07-03

**Authors:** Gilles Riveau, Dominique Deplanque, Franck Remoué, Anne-Marie Schacht, Hubert Vodougnon, Monique Capron, Michel Thiry, Joseph Martial, Christian Libersa, André Capron

**Affiliations:** 1 Inserm – Université Lille 2, Institut Pasteur de Lille, Lille, France; 2 Inserm CIC-CRB 9301, CHRU, Lille, France; 3 Université Lille – Nord de France, Département de Pharmacologie Médicale, Faculté de Médecine, Lille, France; 4 Eurogentec, Parc Scientifique, Seraing, Belgium; The George Washington University Medical Center, United States of America

## Abstract

**Background:**

Treatment of urinary schistosomiasis by chemotherapy remains challenging due to rapid re-infection and possibly to limited susceptibility to praziquantel treatment. Therefore, therapeutic vaccines represent an attractive alternative control strategy. The objectives of this study were to assess the safety and tolerability profile of the recombinant 28 kDa glutathione *S*-transferase of *Schistosoma haematobium* (rSh28GST) in healthy volunteers, and to determine its immunogenicity.

**Methodology:**

Volunteers randomly received 100 µg rSh28GST together with aluminium hydroxide (Alum) as adjuvant (n = 8), or Alum alone as a comparator (n = 8), twice with a 28-day interval between doses. A third dose of rSh28GST or Alum alone was administered to this group at day 150. In view of the results obtained, another group of healthy volunteers (n = 8) received two doses of 300 µg of rSh28GST, again with a 28-day interval. A six-month follow-up was performed with both clinical and biological evaluations. Immunogenicity of the vaccine candidate was evaluated in terms of specific antibody production, the capacity of sera to inhibit enzymatic activity of the antigen, and *in vitro* cytokine production.

**Principal Findings:**

Among the 24 healthy male participants no serious adverse events were reported in the days or weeks after administration. Four subjects under rSh28GST reported mild reactions at the injection site while a clinically insignificant increase in bilirubin was observed in 8/24 subjects. No hematological nor biochemical evidence of toxicity was detected. Immunological analysis showed that rSh28GST was immunogenic. The induced Th2-type response was characterized by antibodies capable of inhibiting the enzymatic activity of rSh28GST.

**Conclusions:**

rSh28GST in Alum did not induce any significant toxicity in healthy adults and generated a Th2-type immune response. Together with previous preclinical results, the data of safety, tolerability and quality of the specific immune response provide evidence that clinical trials with rSh28GST could be continued in humans as a potential vaccine candidate against urinary schistosomiasis.

## Author Summary

Therapeutic vaccines represent an attractive tool in the fight against schistosomiasis. Pre-clinical immunization studies with the schistosome enzyme 28 kDa glutathione *S*-transferase (28GST) has been shown to significantly reduce schistosome egg production and subsequent pathology. The objective of this study was to assess the safety and immunogenicity of the recombinant 28GST of *Schistosoma haematobium* (rSh28GST) in healthy adult volunteers. After three administrations of 100 µg or two of 300 µg, no serious adverse events were reported in the days or weeks after each administration. Some mild adverse events were noted, including minor reactions at the injection site reported for four subjects receiving rSh28GST, but there was no hematological or biochemical evidence of toxicity. Immunological analysis showed that rSh28GST induced a consistent immune response characterized by antibodies endowed with the capacity to inhibit 28GST enzymatic activity. Present data provide evidence that clinical trials with rSh28GST could be continued in humans as a potential vaccine candidate against urinary schistosomiasis.

## Introduction

Schistosomiasis, the second major human parasitic infection after malaria, remains a major health problem in many developing countries, mainly among children and it is estimated that this chronic disease is responsible for 300 000 deaths per year. During *Schistosoma haematobium* infection, morbidity and mortality are associated with worm fecundity and the deposition of schistosome eggs in tissues, especially in the genital and urinary tracts.

More than 20 years after its introduction, the most effective intervention for the control of schistosomiasis remains the use of chemotherapy by praziquantel (PZQ) [1] but, it is generally agreed that this shows numerous limits. Indeed, rapid re-infection following treatment is commonly observed in most endemic areas [2]. Thus, efficient drug delivery requires a substantial infrastructure to regularly cover endemic areas, which makes chemotherapy an expensive approach [3]. In addition, although there is not yet clear evidence of the existence of PZQ-resistant strains, a decreased susceptibility to the drug has been suspected in several countries [4], [5]. The lack of efficient treatment emphasizes the need for more specific and long-term approaches against schistosomiasis. A vaccine strategy may therefore play a crucial role in the control of this parasitic disease.

Among several vaccine candidates [6], the 28 kDa glutathione *S*-transferase antigen (28ShGST) has been well characterized (from molecular cloning to crystallisation) [7], [8]. Immunization of rodents [9], [10], monkeys [11], [12] or cattle [13], [14] with 28GST, followed by experimental infection led to a reduction in worm burden and/or a significant decrease in parasite fecundity, establishing this antigen as a promising vaccine candidate [15], [16]. In addition, field studies have shown that resistance to re-infection in humans could be associated with the presence of acquired anti-28GST antibodies able to inhibit the parasite GST enzymatic activity [17] [18]. Moreover, studies have demonstrated the role of Th2-type responses in anti-schistosome protective immunity in human infection [19].

Toxicological studies were conducted in animal models to support the clinical use of the recombinant Sh28GST (CERB, France and Pasteur Institute of Lille, France; unpublished results). In compliance with Good Laboratory Practice standards, a rabbit model was selected because it produces immune responses to the protein, and the full dose intended for human use can be subcutaneously administered to rabbits. In these conditions, a 4-week study was performed where each rabbit was tested with 9 injections distributed over the lumbar area (2 with aluminium hydroxide (Alum), 2 at a 100-µg dose of rSh28GST in Alum, 2 at a 300-µg dose, 2 at a 1000-µg dose and a final mock injection). A transient local inflammatory reaction after injection was common (erythema, swelling or small induration at the site of injection) and comparable to that seen with Alum alone. In another toxicity study using 3 subcutaneous injections in rats (40-µg dose of rSh28GST in Alum, a 200-µg dose in Alum or Alum alone), animals were monitored for morbidity, mortality, general health and signs of toxicity, food consumption, body weight, clinical observations and also for respiratory, circulatory, dermatological, autonomic and central nervous system parameters. Hematology, blood chemistry, and immunological analyses were also performed. Major tissues and organs were histologically examined on necropsy. In these conditions, no significant clinical, anatomical or biological alterations were found. Finally, a lack of significant alterations was also demonstrated in toxicity studies performed in dogs.

All these findings taken together justify the decision to initiate a phase 1 clinical trial to evaluate the safety and tolerability of the rSh28GST in healthy adult male volunteers. The secondary objective of this randomized phase 1 study was to assess the immunogenicity of rSh28GST.

## Materials and Methods

The study protocol and the patient informed consent form were approved by an independent Ethics Committee (Comité Consultatif de Protection des Personnes dans les Recherches Biomédicales, CCPPRB de Lille, France) and by the French Health competent authorities prior to the start of the study. The study was conducted in the Clinical Investigation Centre of the Lille University Hospital (CIC 9301 CHRU-INSERM, France) in accordance with the Declaration of Helsinki III and with the International Ethical Guidelines for Biomedical Research Involving Human Subjects as laid down by the Council for International Organizations of Medical Sciences in collaboration with the World Health Organization and the Good Clinical Practice guideline CPMP/ICH/135/95 [20] [21] [7].

### Subjects

According to requirements of the French Health authorities regarding first-in-human clinical trials that should avoid the inclusion of children and women with childbearing potential except under specific conditions, the present phase 1 study was conducted in young healthy Caucasian male adults. All subjects had to meet the study inclusion criteria within 21 days prior to treatment, which included written informed consent, age from 18 to 30, absence of any medical history of schistosomiasis, biological parameters (haematological, biochemical, renal and hepatic) within the normal range as well as a normal clinical examination. Moreover, subjects were not smokers and were exempt of any inflammatory or immunological pathology such as atopic diseases, evidence of inflammation or acute infection (including positive serology to viral hepatitis B and C or HIV), any immunological deficiency, any clinically relevant alcohol or drug use (cannabis, opiates, cocaine, amphetamines, benzodiazepines, nicotine, barbiturates, meprobamates or antidepressant drugs according to a urinary drug and metabolites screen) or any other medication use within 2 weeks before the study, any vaccination within the last 6 months and had no antibodies against the Sh28GST protein.

### Study design

**Figure 1:**
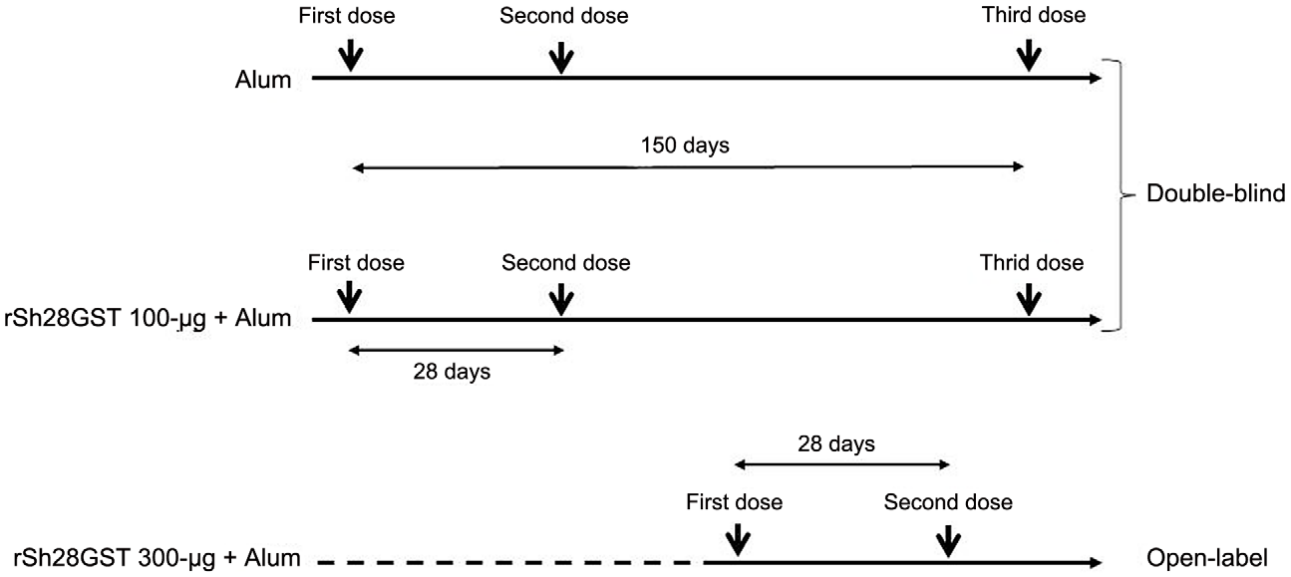
Study design.

This phase 1 randomized controlled study was designed to investigate the safety and tolerability of rSh28GST antigen injected subcutaneously (deltoid muscle) with Alum as adjuvant. In a first step, the study was performed on healthy male volunteers according to a double-blind design. Subjects selected at random received either two 100 µg doses of rSh28GST together with Alum as vaccine adjuvant (n = 8) or two doses of Alum in a 0.5 ml solution as a comparator (n = 8) with a 28-day interval (Day 0 [D_0_] and Day 28 [D_28_]) between doses in both groups (figure 1). These subjects also received a third administration of 100 µg Sh28GST or Alum alone on day 150 [D_150_]. As a dose escalation trial, a third group of healthy volunteers received two 300 µg doses of rSh28GST at a 28-day interval according to an open-label design (figure 1). All biological analyses were carried out blind to treatment conditions.

### Treatment

Batches of rSh28GST were produced and purified from recombinant *Saccharomyces cerevisiae* culture (TGY73.4 - pTG8889 strain) under Good Manufacturing Practice (GMP) conditions by Eurogentec S.A. (Belgium). The rSh28GST clinical batch (# B98H11) was conserved lyophilized (124 µg per vial for the administrated dose of 100 µg; 352 µg per vial for the administered dose of 300 µg) by Sterilyo (France) under GMP conditions. The lyophilized preparation was re-suspended extemporaneously using 0.6 ml of apyrogenic and sterile aluminium hydroxide solution 0,2% (Al_2_O_3_ 0.2%; Al(OH)_3_ 3%; NaCl 9 g/L; ammonium carbonate buffer 10 mM, pH7.8) (Alum from Superfos, Denmark; batch #14093) and administered in a volume of 0.5 ml.

### Assessment of clinical tolerability

Following each administration, participants were kept under constant observation during 4 hours and further evaluated at D_1_, D_21_, D_28_, D_29_, D_49_, D_120_, D_150_, D_165_ and D_180_. Clinical evaluations consisted of measurement of vital signs and assessment of the local injection site and general signs or symptoms. Local signs and symptoms included pain or tenderness, swelling, induration and erythema at the site of injection. Assessment of general tolerability was obtained through a complete physical examination including an examination of general appearance, body weight and rectal temperature, head, eyes, ears, nose and throat, neck, skin, cardiovascular and respiratory system, abdominal system, nervous system, lymphatic area, blood pressure, pulse and respiratory rates. Recordings of 12-lead ECG were also obtained allowing analysis of ventricular rate, rhythm, PR interval, QRS duration and QT/QTc. A number of general signs and symptoms were specially considered including fever (rectal temperature ≥38°C), headache, nausea, vomiting, myalgia, arthralgia, irritability/fussiness and drowsiness, loss of appetite and sleep disturbances. In addition to these predefined signs and symptoms, all adverse events were reported throughout the study. Adverse events were graded by severity following FDA guidance [22] and judged for potential association with vaccination. Briefly, grade 1 adverse events were easily tolerated, causing minimal discomfort and not interfering with daily activities; grade 2 adverse events were sufficiently discomforting to interfere with normal activities, grade 3 adverse events prevented normal daily activities while grade 4 were potentially life threatening [22].

### Assessment of biological tolerability

Biological tolerability was assessed at baseline and at D_1_, D_21_, D_28_, D_29_, D_49_, D_120_, D_150_, D_165_ and D_180_ using clinical laboratory tests including haemoglobin, haematocrit, packed cell volume, mean corpuscular volume, mean cell haemoglobin, mean cell haemoglobin concentration, white blood cells and platelet counts; coagulation tests such as activated partial thromboplastin time (PTT), prothrombin time and International Normalized Ratio (INR); serum biochemistry such as sodium, potassium, creatinine, urea, alanine amino transaminase (ALT), aspartate amino transaminase (AST), gamma glutamine transferase (gamma-GT), bilirubin, uric acid, total protein, alkaline phosphatases, calcium, C-reactive Protein (CRP) and fasting glucose; urinalysis including pH, protein, glucose, ketones, urobilirubin, blood and leucocytes. For all these laboratory tests, toxicity grading was assigned using local laboratory values or previously published normal reference ranges [23].

### Assessment of immunogenicity

Specific antibody titers in individual sera were determined by ELISA. rSh28GST (10 µg/ml) was coated on 96-well plates (Nunc, Roskilde, Denmark) 2h30 at 37°C. Plates were blocked with phosphate-buffered saline containing 0.5% gelatin (Merck, Darmstadt, Germany). Then, six serial dilutions of individual sera (first dilution for IgG1 1/100; IgG2, IgG3 and IgA 1/20; IgG4 and IgE 1/10) were added and incubated. Each corresponding biotinylated mAbs to human Ig isotype (Southern Biotechnology, Birmingham, AL, USA) was incubated (2 h at 37°C) at a 1/1000 dilution for IgG, IgG1, IgG2, IgG3, IgG4, IgE and IgA, and 1/1500 for IgG1 detection. Peroxidase-conjugated streptavidin (1/1500; 30 min at 37°C) was then added (Amersham, Les Ulis, France). Colorimetric development was performed with ABTS (Sigma, St. Louis, MO, USA) in 50 mM citrate buffer (pH = 4) and absorbance (OD) was measured at 405 nm. Titers were defined as the highest dilution yielding an absorbance value three times above the background (wells containing buffer instead of serum in the same plate). Vaccinated individuals were considered as positive responders when their titer was greater than three fold the standard deviation above the mean titer value of all individuals at D_0_ (vaccine and comparator group).

Cytokine production was evaluated in whole blood diluted in RPMI 1640 medium (Gibco, Courbevoie, France) to obtain 2×10^6^ mononuclear cells per ml. The blood preparation (1 ml) was then plated in duplicate culture in 24 flat-bottomed plates (Nunc) and stimulated with 1 ml of rSh28GST antigen (20 µg/ml). Supernatants were harvested on day 3 for analysis of cytokine production. Quantitative ELISA for human IL-2, IL-4, IL-5, IL-10, IL-12, IL-13 and IFN-γ detection were performed in supernatants using EASIA Kits (Immunotech, Marseille, France) and human TGF-β1 by ELISA System Kit (Promega, Madison, WI, USA). Cytokine concentrations were expressed as pg/ml or IU/ml (for IFN-gamma) after subtracting the amount detected in unstimulated control cultures. Cytokine production was considered stimulated when the value was more than three times the standard deviation above the mean value.

To evaluate the capacity of induced antibodies to inhibit GST activity, 20 µl of rSh28GST solution (4 µg/ml in 50 mM potassium phosphate at pH 6.5) was incubated with 20 µl of human serum for 1 h at 37°C in Immulon 3 Plates (Nunc, Roskilde, Denmark). The enzymatic reaction was carried out using 1-chloro-2,4, dinitrobenzene (Sigma, St. Louis, MO) substrate as described elsewhere [18]. Enzymatic reaction intensity was measured by OD at 340 nm. Every enzyme-inhibition test was performed with appropriate controls (enzyme without serum and tested serum alone). The percentage of inhibition was calculated by the ratio of GST activity after serum incubation to the GST activity control. Percentage inhibition above 10% was considered significant.

### Statistical analysis

The sample size for this first-in-human study was not powered on the basis of statistical hypothesis testing since as is customary for phase 1 studies, hypothesis testing is not part of the primary objective. The target number of subjects was expected to minimize the risk by not exposing too large a population to treatment, while providing an ample size to gain information on safety and tolerability. The sample size of 8 subjects for each group (both rSh28GST and control) ensured at least an 85% probability of observing ≥1 subject with a specific adverse event if the background incidence rate of that adverse event was ≥10%. This sample size also ensured a ≥80% probability of observing common placebo-associated adverse events, defined as those events with a population incidence ≥15%. Descriptive statistics are presented using mean and standard deviation or median and range as required. Baseline laboratory values were defined as the pre-treatment values obtained during the baseline screening visit. Statistical analysis for the immunological studies was performed using the Wilcoxon signed rank test to assess the difference in immunological parameters between days using SPSS v15 for Windows. Differences were considered significant at *p*<0.05.

## Results

### Subject characteristics

A total of 31 healthy male volunteers underwent initial evaluation for the study. Among these, 7 were eliminated using exclusion criteria such as allergy or hypersensitivity, tobacco use or elevated immunoglobulins. Finally, 24 volunteers were randomized. They were 18 to 29 years old (23±3 years), height from 170 to 191 cm (178±6 cm) and weight from 52 to 82 kg (71±8 kg). All other clinical and routine laboratory data were considered in normal ranges although 1 subject in the comparator group and 3 subjects in the 300 µg group had a slight increase in bilirubin according to local laboratory values (main baseline biological data in table 1). All volunteers were followed until the end of the study. All local, general and biological adverse events reported in the present study are detailed in table 2.

**Table 1:** Main baseline laboratory results.

Component	Placebo	rSh28GST 100 µg	rSh28GST 300 µg	Reference ranges
WBC, ×10^3^/µL	5.79±1.58	5.88±1.10	5.70±0.65	4.5–11.0
Hemoglobin, g/dL	15.54±0.74	15.40±0.68	15.59±0.84	13.5–17.5
Platelet, ×10^3^/µL	234.29±38.67	218.50±39.78	230.00±48.87	150–350*
PTT, sec	35.59±2.36	34.14±4.07	36.79±5.31	25–40*
Glucose, g/L	0.91±0.07	0.90±0.09	0.88±0.04	0.75–1.15
Urea, g/L	0.34±0.09	0.36±0.07	0.26±0.04	0.15–0.45*
Creatinine, g/L	10.13±0.83	11.50±0.93	10.75±0.89	<15
ALT, UI/L	17.50±4.87	12.38±5.83	11.88±9.58	7–45*
AST, UI/L	16.00±2.62	15.75±3.01	15.13±4.16	7–30*
Bilirubin, mg/L	7.50±3.02	8.13±3.68	11.25±4.13	1–12*

Abbreviations: WBC, white blood cell count; PTT, partial thromboplastin time; ALT, alanine aminotransferase; AST, aspartate aminotransferase. Data are presented as mean ± standard deviation.

*: Local laboratory values. All other biological tests were in normal ranges.

**Table 2:** Main adverse events reported in the course of each administration.

	Aluminium hydroxide			rSh28GST 100 µg			rSh28GST 300 µg	
	n = 8			n = 8			n = 8	
	1st dose	2nd dose	3rd dose	1st dose	2nd dose	3rd dose	1st dose	2nd dose
**Local signs**								
Swelling/induration	0	0	0	0	1 (G1)	1 (G1)	2 (G1)	1 (G1)
Erythema	0	0	0	0	2 (G1)	1 (G1)	1 (G1)	1 (G1)
**General signs**								
Fever	0	0	0	1 (G1)	1 (G2)‡	0	0	0
Headache	0	1 (G1)	0	0	0	0	0	0
Diarrhea	0	0	0	0	1 (G2)‡	0	0	0
Other illness	0	1 (G3)*	0	2 (G1)	1 (G3)¥	0	2 (G1, G3)†	0
**Biological signs**								
PTT increase	0	0	0	0	1 (G2)‡	0	1 (G1)	0
Bilirubin increase	1 (G1)	2 (G1, G2)	2 (G1, G2)	2 (G1)	2 (G1)	0	1 (G1)	2 (G1)

G, grade.

*: Severe bilateral leg erythema unrelated to treatment.

**†:** Flu-like syndrome or rhinitis (one subject in the Sh28GST 100 µg group).

**‡:** Same patient with febrile diarrhea.

**¥:** Pharyngitis requiring antibiotherapy. No other adverse events were reported. See text for details.

### Safety and tolerability

#### Clinical adverse events

In the hours following the first administration, slight local swelling and erythema were reported in only 2 subjects that received rSh28GST at the 300 µg dose while no systemic clinical adverse reactions occurred in any other subject (table 2). After the second administration, grade 1 local signs were newly observed in 2 subjects from the rSh28GST 100 µg group while they occurred again in 1 subject from the rSh28GST 300 µg group. Finally, only 1 subject in the rSh28GST 100 µg group again experienced local signs after the third and last administration. In terms of general signs, 3 subjects (1 in the 100 µg group and 2 in the 300 µg group) experienced a moderate flu-like syndrome between D_0_ and D_28_, leading to antibiotic treatment for one (grade 3) while another reported a transient rhinitis. These symptoms disappeared before the D_28_ visit except for one subject (rSh28GST 100 µg group) who still presented hyperthermia (38.7°C, grade 2) with moderate cough that spontaneously resolved during the following week. For this subject, the second administration was performed one week later (D_35_). In the comparator group, one participant suffered important erythema on internal parts of both legs in the evening following the second administration (grade 3). This erythema was finally not related to adjuvant administration since it occurred after an intensive and prolonged motorbike ride during the days before injection. Four other participants presented weak side effects while 2 subjects from the rSh28GST 100 µg group suffered from other illnesses: rhinitis with fever that required antibiotic treatment for one (grade 3) and transient febrile diarrhea 2 weeks after the second vaccine administration that rapidly resolved with symptomatic treatment (grade 2) (table 2).

#### Biological adverse events

No major biological adverse event occurred in the course of rSh28GST administration. A slight PTT elevation was observed in one participant at D_21_, which led to the diagnosis of a moderate familial form of Von Willebrand disease. This disorder was undetected prior to the study and curiously PTT remained strictly normal at all other visits. A transient PTT elevation (grade 2) associated with anticoagulant circulating antibodies was also reported in the subject suffering from febrile diarrhea but this biological abnormality spontaneously and rapidly resolved. After the first vaccine administration, a slight bilirubin increase occurred in 1 subject in the comparator group and 1 subject in the rSh28GST 300 µg group, both subjects that already had a slight increase in bilirubin at baseline. An increase in bilirubin level also newly occurred in 2 other subjects in the rSh28GST 100 µg group (table 2). After the second administration, a total of 6 subjects experienced a clinically insignificant, slight increase in bilirubin level (2 subjects in each group including the comparator) (table 2). In 3 subjects this adverse event occurred for the first time while it occurred for a second time in 2 subjects (rSh28GST 100 µg or comparator group), both subjects that already had a slight increase in bilirubin at baseline. Such a biological abnormality at baseline seems not necessarily to be a marker for a future bilirubin increase since 2 subjects from the rSh28GST 300 µg group that also had a slight increase in bilirubin at baseline remained stable during the whole course of the study. Finally, after the third administration, the only biological adverse event was again a slight and not clinically significant increase in bilirubin in 2 subjects from the comparator group (table 2). One of these subjects presented a slight increase in bilirubin since baseline analysis, an abnormality that finally completely returned to normal values at D_120_.

### Immunological responses

#### Antibody response to rSh28GST

In Tables 3 and 4 are presented results of specific antibody responses after administration of 100 µg rSh28GST in Alum (table 3) and comparator (table 4). A specific IgG1 response was predominant and significantly induced after the first administration of rSh28GST in 6 out of 8 vaccinated volunteers at D_21_. The mean IgG1 titer strongly increased at D_49_, 21 days after the second injection (P<0.05) and all vaccinated adults were positive responders (table 3). Analysis of other anti-rSh28GST isotypes showed low levels of specific IgG2 and IgG4 antibodies whereas the IgG3 response was high and significantly induced in 7/8 adults at D_49_. At this time point, mean IgE and IgA titers were not significantly different from values obtained at D_0_. A consistent immune response to rSh28GST was recorded during 4 months after the second administration (D_49_/D_150_). After a third administration of rSh28GST (D_150_), a significant increase in the specific antibody response including all isotypes, IgE excepted, was detected (D_165_ and D_180_ compared to D_150_; P<0.05). Following administration of Alum, no subject from the comparator group showed a specific antibody response against rSh28GST throughout the trial duration (table 4). The specific antibody response observed in volunteers receiving twice the 300 µg dose of rSh28GST was not significantly different to that obtained after two administrations of 100 µg (data not shown).

**Table 3:** Anti-Sh28GST antibody levels in healthy adult volunteers after administration of rSh28GST 100 µg.

Day	IgG1^a^	IgG2	IgG3	IgG4	IgE	IgA1
D_0_ b (n = 8)	65±21	4±3	18±16	0	4±2	22±18
D_21_ (n = 8)	640±159* (7)	5±5	49±31 (3)	0	0	26±16
D_28_ b (n = 8)	658±151* (7)	7±6	63±33* (3)	0.6±0.6	1±1	21±17
D_49_ (n = 8)	2249±264* (8)	50±22* (4)	277±53* (8)	21±19 (1)	0	38±20* (4)
D_120_ (N = 8)	2613±464* (8)	89±30* (4)	212±64* (6)	121±28* (7)	1±1	28±20 (2)
D_150_ b (n = 6)	2424±522* (6)	71±36* (3)	161±68* (4)	134±40* (5)	0	29±23
D_165_ (n = 6)	8825±3115** (6)	220±59** (6)	323±98* (6)	391±63** (6)	2±2	90±28** (5)
D_180_ (n = 6)	11810±5061** (6)	262±63** (5)	277±79* (6)	797±294** (6)	0	63±23** (4)

a Specific Ig antibody levels are expressed as the mean of individual titer (± SEM). Number of responders after vaccination is indicated in parentheses.

b Day of Sh28GST administration (D_0_; D_28_; D_150_).

*: Significantly different (P<0.05) compared to D_0_ (comparison of mean using the Wilcoxon test).

**: Significantly different (P<0.05) compared to D_150_ (comparison of mean using the Wilcoxon test).

**Table 4:** Anti-Sh28GST antibody levels in healthy adult volunteers after administration of Alum (comparator group).

Day	IgG1^a^	IgG2	IgG3	IgG4	IgE	IgA1
D_0_ b (n = 8)	42±18	0	16±9	0	0	10±4
D_21_ (n = 8)	32±18	0	4±2	0	0	5±2
D_28_ b (n = 8)	37±19	0	10±7	0	0	15±8 (1)
D_49_ (n = 8)	35±16	0	12±8	0	3±2	8±3
D_120_ (N = 8)	38±18	0	10±7	0	0	11±4 (2)
D_150_ b (n = 7)	31±15	0	8±8	0	1±1	6±3
D_165_ (n = 7)	33±15	0	10±8	0	2±2	11±5 (1)
D_180_ (n = 7)	41±19	0	11±8	0	2±1	9±5 (1)

a Specific Ig antibody levels are expressed as the mean of individual titer (± SEM). Number of responders after vaccination is indicated in parentheses.

b Day of comparator administration (D_0_; D_28_; D_150_).

#### Specific induction of cytokines

Measurements of IL-2, IL-10, IL-12, IFN-γ and TGF-β after *in vitro* stimulation of mononuclear cells by rSh28GST are presented in table 5. Stimulation of cytokine production was not detected at any time in individuals from the comparator group (data not shown).

**Table 5:** *In vitro* specific production^a^ of cytokines after stimulation of mononuclear cells in vaccinated adults (after 2 administrations).

Days	IL-2 (pg/ml)	IL-5 (pg/ml)	IL-10 (pg/ml)	IL-12 (pg/ml)	IL-13 (pg/ml)	IFN-γ (IU/ml)	TGF-β (pg/ml)
D_0_ (n = 8)	2.4±3.6	0	32.1±34.7	0.3±0.6	0	0.09±0.13	13.5±20.1
D_49_ (n = 8)	25.7±25.9 (5)	33.7±28.9* (6)	22.7±16.8 (0)	3.1±4.7 (0)	22.3±13.6* (6)	0.47±0.53 (1)	8.8±13.2 (0)

a Cytokine production is expressed as the mean (± SEM) of individual specific production (after subtracting the amount detected in unstimulated control cultures at each day and for each individual). Number of responders is indicated in parentheses.

*: Significantly different (P<0.05) compared to D0 (comparison of mean using the Wilcoxon test).

In volunteers receiving 100 µg rSh28GST, means of IL-2, IL-10, IL-12, IFN-γ and TGF-β production after *in vitro* antigen stimulation were not significantly different at D_49_ compared to D_0_, although 5/8 and 1/8 adults were positive responders for IL-2 and IFN-γ production respectively. In contrast, production of IL-5 and IL-13 was significantly induced after two administrations of rSh28GST 100 µg in 6 of the 8 vaccinated adults.

#### Inhibition of Sh28GST enzymatic activity

**Figure 2:**
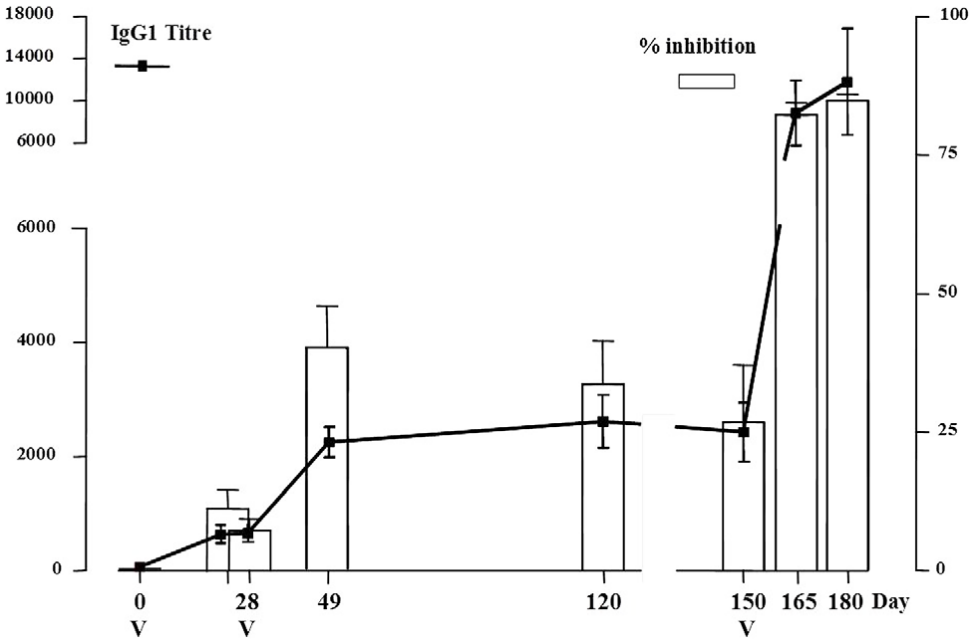
Specific IgG1 production and inhibition of enzymatic activity of rSh28GST by sera of vaccinated volunteers. Adult human volunteers received three administrations of rSh28GST of 100 µg respectively at D0, D28 and D150. IgG1 production is expressed as mean +/− SEM of titers at each time. Titer was defined as the highest dilution yielding an absorbance three times above background. Enzymatic inhibition is expressed in % (histograms). Percentage of inhibition was calculated by the ratio GST activity after serum incubation to GST activity control. This value was considered significantly positive above 10%.

The capacity of individual sera to inhibit rSh28GST enzymatic activity is presented in Fig. 2. Significant inhibition was observed in 4/8 vaccinated volunteers after the first 100 µg administration (*p*<0.05 compared to D_0_) whereas sera from all vaccinated subjects showed significant inhibition after the second injection (D_0_/D_49_; *p*<0.05). In addition, a significant increase in rSh28GST inhibition in sera from all vaccinated subjects was recorded after the third administration (D_165_ and D_180_; *p*<0.05). A correlation of inhibitory capacity with the production of specific isotypes was obtained with IgG1 and IgG3. No significant inhibition was observed with sera obtained from the comparator group at any time of the study. The observed inhibitory capacity of sera after two administrations of the 300 µg dose of rSh28GST was similar to those obtained from volunteers receiving twice the 100 µg dose (data not shown).

## Discussion

The primary objective of this phase 1 clinical trial was the demonstration of the innocuity of vaccination with recombinant *S. haematobium* 28 kDa glutathione *S*-transferase using Alum as an adjuvant. Previous pre-clinical trials of this candidate vaccine in a variety of animal models had shown that it was particularly effective in reducing parasite egg-laying, egg viability and pathology, justifying its potential to reduce disease morbidity in the target populations. We had also shown that administration of the proposed vaccine dose to rabbits, as well as supplementary studies in rats and dogs, caused no significant clinical, anatomical or biological alterations.

Although the target population in endemic areas will be school age children of both sexes, ethical considerations (see Methods) dictated that this phase 1 trial should be carried out on adult male subjects. The overall conclusion of the study is that no severe side-effects were detected (the study design allowed us to exclude adverse events that were unrelated to the vaccination) and that only very few minor, clinically insignificant adverse effects were caused by the vaccination. Some mild (grade 1) adverse effects, restricted to local swelling and erythema around the injection site, were reported in 4 subjects out of the 16 receiving rSh28GST. These effects were reiterated in only one subject and were not seen in the comparator group receiving Alum alone.

Several of the observed adverse reactions may have trivial explanations, not related to vaccine administration. For instance, the episode of rhinitis occurred in winter and may therefore be seasonal and not caused by administration of Sh28GST. The extensive leg erythema noted in another subject was the consequence of a prolonged motorbike ride. The two instances of increases in PTT were again probably unrelated to the vaccination: in one case concomitant with an episode of febrile diarrhoea and in the other case caused by a previously undiagnosed familial form of Von Willebrand disease.

The most frequently observed adverse event detected was a slight increase in bilirubin levels occurring in 8 patients. This was independent of the treatment received since patients receiving rSh28GST or Alum alone were affected. Sh28GST is therefore unlikely to be incriminated in this effect, particularly as a number of subjects already had an elevated level of bilirubin before the start of the study. Even though there is evidence that aluminium hydroxide may be highly concentrated in the liver, the long-term use of this adjuvant provide no evidence of hepatotoxicity in humans [24]. Moreover, it is possible that small variations in the assay used by the local laboratory may have contributed to these observations.

A secondary objective of this study was to evaluate the immunogenicity of rSh28GST in humans, particularly in terms of the antibody response. Both doses used were effective in generating a specific antibody response that was detected in all patients after the second dose. Moreover, increasing the dose (from 100 to 300 µg) had no effect on the antibody titer obtained. The isotype profile was characterized by the predominance of the IgG1 subclass both after the first and subsequent injections, whereas low levels of IgG2 and IgG3 were recorded after the second administration. This isotype profile suggests that the vaccine formulation in Alum induced a Th2-type immune response [25] that is typical of the immune orientation induced by this adjuvant [26]. This is further supported by the cytokine production after *in vitro* stimulation with rSh28GST of cultured mononuclear cells from immunized patients. This showed a high level of the Th2-type cytokines IL-5 and IL-13 [27] after the second vaccine administration. However, despite this profile it is important to note that no IgE antibodies specific for rSh28GST were generated using the vaccination protocol employed in this study.

In this phase I trial, a crucial aspect of the immune response generated is the inhibition of rSh28GST enzymatic activity by antibodies from vaccinated adults after two administrations. This result demonstrated that the specific antibody response induced after rSh28GST/alum administration in humans was endowed with the biological activity previously described to be related to the anti-fecundity effect of 28GST, which is able to prevent the development of the schistosomiasis pathology in experimental models [15], [28].

### Conclusion

This phase 1 study demonstrates the safety, tolerability and immunogenicity of rSh28GST in Alum as a first vaccine candidate against schistosomiasis. These results allowed us to conduct further clinical trials in endemic regions (manuscripts in preparation). The objectives of these studies were again to assess the safety, tolerability and immunogenic profile of the recombinant rSh28GST in Alum in healthy school children, and also in adults and children infected with *S. haematobium*. A clinical trial evaluating the efficacy of the vaccine candidate is currently in progress in a large cohort of infected schoolchildren.

## References

[1] Redman CA, Robertson A, Fallon PG, Modha J, Kusel JR (1996). Praziquantel: An urgent and exciting challenge.. Parasitology Today.

[2] Stelma FF, Talla I, Sow S, Kongs A, Niang M (1995). Efficacy and Side Effects of Praziquantel in an Epidemic Focus of Schistosoma mansoni.. The American Journal of Tropical Medicine and Hygiene.

[3] Bergquist NR, Colley DG (1998). Schistosomiasis Vaccine:Research to Development.. Parasitology Today.

[4] Fallon PG, Sturrock RF, Capron A, Niang M, Doenhoff MJ (1995). Short Report: Diminished Susceptibility to Praziquantel in a Senegal Isolate of Schistosoma mansoni.. The American Journal of Tropical Medicine and Hygiene.

[5] Sabra AN, Botros SS (2008). Response of Schistosoma mansoni isolates having different drug sensitivity to praziquantel over several life cycle passages with and without therapeutic pressure.. Journal of Parasitology.

[6] Capron A, Riveau GJ, Bartley PB, McManus DP (2002). Prospects for a schistosome vaccine.. Current Drug Targets Immune, Endocrine and Metabolic Disorders.

[7] Balloul JM, Sondermeyer P, Dreyer D, Capron M, Grzych JM (1987). Molecular cloning of a protective antigen of schistosomes.. Nature.

[8] Trottein F, Vaney M-C, Bachet B, Pierce R-J, Colloc'h N (1992). Crystallization and preliminary X-ray diffraction studies of a protective cloned 28 kDa glutathione S-transferase from Schistosoma mansoni.. Journal of Molecular Biology.

[9] Xu CB, Verwaerde C, Gras-Masse H, Fontaine J, Bossus M (1993). Schistosoma mansoni 28-kDa glutathione S-transferase and immunity against parasite fecundity and egg viability. Role of the amino- and carboxyl-terminal domains.. Journal of Immunology (Baltimore, Md: 1950).

[10] Dupré L, Herv M, Schacht AM, Capron A, Riveau G (1999). Control of schistosomiasis pathology by combination of Sm28GST DNA immunization and praziquantel treatment.. The Journal of Infectious Diseases.

[11] Boulanger D, Reid GD, Sturrock RF, Wolowczuk I, Balloul JM (1991). Immunization of mice and baboons with the recombinant Sm28GST affects both worm viability and fecundity after experimental infection with Schistosoma mansoni.. Parasite Immunology.

[12] Boulanger D, Warter A, Trottein F, Mauny F, Brémond P (1995). Vaccination of patas monkeys experimentally infected with Schistosoma haematobium using a recombinant glutathione S-transferase cloned from S. mansoni.. Parasite Immunology.

[13] De Bont J, Vercruysse J, Grzych JM, Meeus PF, Capron A (1997). Potential of a recombinant Schistosoma bovis-derived glutathione S-transferase to protect cattle against experimental and natural S. mattheei infection.. Parasitology.

[14] Grzych JM, De Bont J, Liu J, Neyrinck JL, Fontaine J (1998). Relationship of impairment of schistosome 28-kilodalton glutathione S-transferase (GST) activity to expression of immunity to Schistosoma mattheei in calves vaccinated with recombinant Schistosoma bovis 28-kilodalton GST.. Infection and Immunity.

[15] Riveau G, Capron A (1996). Vaccination against schistosomiasis: concepts and strategies. Concepts in Vaccine Design.

[16] Capron A, Riveau G, Capron M, Trottein F (2005). Schistosomes: the road from host-parasite interactions to vaccines in clinical trials.. Trends in Parasitology.

[17] Grzych JM, Grezel D, Xu CB, Neyrinck JL, Capron M (1993). IgA antibodies to a protective antigen in human Schistosomiasis mansoni.. Journal of Immunology (Baltimore, Md: 1950).

[18] Remoué F, Rogerie F, Gallissot MC, Guyatt HL, Neyrinck JL (2000). Sex-dependent neutralizing humoral response to Schistosoma mansoni 28GST antigen in infected human populations.. The Journal of Infectious Diseases.

[19] Dunne DW, Hagan P, Abath FG (1995). Prospects for immunological control of schistosomiasis.. Lancet.

[20] WHO (1995). Guidelines for Good Clinical Practice (GCP) for Trials on Pharmaceutical Products - Sixth Report..

[21] WHO (1996). Guidelines for Good Clinical Practice (3 errata post step 4 included)..

[22] FDA (2007). Guidance for Industry: Toxicity grading scale for healthy adult and adolescent volunteers enrolled in preventive vaccine clinical trials..

[23] Kratz A, Ferraro M, Sluss PM, Lewandrowski KB (2004). Case records of the Massachusetts General Hospital. Weekly clinicopathological exercises. Laboratory reference values.. The New England Journal of Medicine.

[24] Fiejka M, Fiejka E, Dougaszek M (1996). Effect of aluminium hydroxide administration on normal mice: tissue distribution and ultrastructural localization of aluminium in liver.. Pharmacology & Toxicology.

[25] Romagnani S (1996). Development of Th 1- or Th 2-dominated immune responses: what about the polarizing signals?. International Journal of Clinical & Laboratory Research.

[26] Gupta RK, Rost BE, Relyveld E, Siber GR (1995). Adjuvant properties of aluminum and calcium compounds.. Pharmaceutical Biotechnology.

[27] Romagnani S, Parronchi P, D'Elios MM, Romagnani P, Annunziato F (1997). An update on human Th1 and Th2 cells.. International Archives of Allergy and Immunology.

[28] Capron A (1998). Schistosomiasis: forty years' war on the worm.. Parasitology Today (Personal Ed).

